# Sleep Quality and Bariatric Surgery—Can We Treat Sleep Disturbances and Insomnia in Patients with Obesity with Laparoscopic Sleeve Gastrectomy?

**DOI:** 10.3390/jcm13164820

**Published:** 2024-08-15

**Authors:** Krzysztof Wyszomirski, Antonina Ślubowska, Jan Dębski, Klaudia Skibiak, Józef Przybyłowski, Maria Czerwińska, Maciej Walędziak, Anna Różańska-Walędziak

**Affiliations:** 1 Department of Human Physiology and Pathophysiology, Faculty of Medicine, Collegium Medicum, Cardinal Stefan Wyszynski University in Warsaw, 01-938 Warsaw, Poland; kj.wyszomirski@gmail.com (K.W.); aniaroza@tlen.pl (A.R.-W.); 2 Department of Biostatistics and Research Methodology, Faculty of Medicine, Collegium Medicum, Cardinal Stefan Wyszynski University in Warsaw, 01-938 Warsaw, Poland; a.slubowska@uksw.edu.pl; 3 Faculty of Medicine, Collegium Medicum, Cardinal Stefan Wyszynski University in Warsaw, 01-938 Warsaw, Poland; jdebski@student.uksw.edu.pl (J.D.); k.skibiak@student.uksw.edu.pl (K.S.); j.przybylowski@student.uksw.edu.pl (J.P.); mariaczerwinska@student.uksw.edu.pl (M.C.); 4 Department of General, Oncological, Metabolic and Thoracic Surgery, Military Institute of Medicine, Szaserów 128 St., 04-141 Warsaw, Poland

**Keywords:** sleep quality, sleep disturbances, obesity, bariatric surgery, insomnia, snoring, early morning awakenings, eating at night

## Abstract

**Introduction:** Bariatric surgery is the mainstay of treatment of obesity, with a proven, long-lasting effect on body weight reduction and remission of co-morbidities. Sleep disorders, including insomnia, and deteriorated sleep quality and duration are associated with obesity, and a reduction in body weight can be associated with a reduction in prevalence of sleep disorders. The purpose of this study was to assess the influence of laparoscopic sleeve gastrectomy (LSG) on the prevalence and intensity of different sleep disturbances. **Methods:** This observational prospective study included 80 patients qualified for bariatric surgery who filled in a questionnaire with a set of structured questions about different sleep disturbances, such as difficulties in falling asleep, night awakenings, early morning awakenings, snoring, and nightmares, as well as eating at night and daytime dysfunction, supplemented with Athens Insomnia Scale (AIS), before and 6 months after bariatric surgery. **Results:** There was a statistically significant reduction in incidence of night awakenings, with 40.00% of participants reporting night awakenings before surgery and, respectively, 25.00% after surgery. A significant reduction was also observed in the rate of patients who reported snoring, with 60.00% before the surgery and 38.75% after the surgery (*p* < 0.05). There was a correlation present between estimated weight loss % (EWL%) and reduction in snoring (*p* < 0.05). The mean total AIS score before surgery was 7.21 and 5.99 after surgery, and the change was statistically significant (*p* < 0.05). A total AIS score of 8 or more, the cutoff score for insomnia diagnosis according to the Polish validation of the Athens Insomnia Scale, was present in 44.16% of cases before surgery and in 38.00% after surgery (*p* = 0.52). There was a significant difference in the incidence of awakening during the night score before and after surgery (*p* < 0.05; CI 0.022–0.341), sleep quality (*p* < 0.05; CI 0.0105–0.4311), well-being during the day (*p* < 0.05; CI 0.0273–0.4143), and sleepiness during the day (*p* < 0.05; CI 0.101–0.444). **Conclusions:** LSG is observed to have a positive effect on selected sleep disturbances and insomnia remission in patients with obesity, measured by a significant reduction in Athens Insomnia Scale scores in follow-up 6 months after surgery. Additionally, patients after bariatric surgery reported less night awakenings and there was a lower rate of snoring. Therefore, LSG can be considered an effective therapeutic tool for insomnia in patients with obesity.

## 1. Introduction

Excess body weight is an emerging health problem worldwide. Recent reports presented by the World Health Organization show that in 2022, over 2.5 billion adults had excess body weight and almost 1 billion were obese. The prognoses carried out by numerous associations are consistent and pessimistic—further aggravation of the excess body weight problem will be observed in the next years to come [1]. The complications of obesity are commonly known, including cardiovascular co-morbidities, type 2 diabetes mellitus (T2D), elevated risk of certain malignancies, and quality of life deterioration [2,3,4]. Additionally, obesity and its co-morbidities are a growing economic problem for healthcare systems [5].

Bariatric surgery (BS) is the mainstay of treatment of obesity, with a proven, long-lasting effect on body weight reduction and the remission of co-morbidities [6,7,8]. Bariatric surgery is proven to have high efficacy in reducing the prevalence and intensity of obstructive sleep apnea (OSA) in patients with obesity [9,10,11,12,13]. Body mass index (BMI) remains the most applicative indicator to assess the level of obesity. Both previous and current international guidelines use BMI as the most important parameter to establish indications for bariatric surgery. According to guidelines presented by National Institute of Health (NIH), there are two main cutoff points over which bariatric surgery is indicated—BMI ≥ 40 kg/m^2^ and BMI ≥ 35 kg/m^2^—with co-morbidities, the remission of which are expected after bariatric surgery: “*T2D, hypertension, dyslipidemia, obstructive sleep apnea, cardiovascular disease (e.g., coronary artery disease, heart failure, atrial fibrillation), asthma, fatty liver disease and nonalcoholic steatohepatitis, chronic kidney disease, polycystic ovarian syndrome, infertility, gastroesophageal reflux disease, pseudotumor cerebri, and bone and joint diseases*” [14]. There is a trend in recent years to lower the threshold for qualification to bariatric surgery—in 2022, American Society of Metabolic and Bariatric Surgery (ASMBS) and International Federation for the Surgery of Obesity and Metabolic Disorders published recommendations for lowering BMI value to 30 kg/m^2^ and 35 kg/m^2^, respectively, based on numerous preceding studies that had proven the high efficacy and safety of bariatric surgery [3,14].

Undisturbed sleep is one of the fundaments of health and quality of life. However, the prevalence of sleep disorders has increased in recent years. Sleep disorders include OSA, hypoventilation syndrome and various sleep disturbances, difficulties in falling asleep, night awakenings, early morning awakenings, snoring, and nightmares, as well as insomnia. The most common, widely used, and validated diagnostic tool for insomnia is the Athens Insomnia Scale. The AIS score can be used both for the diagnosis of insomnia and the evaluation of its intensity. Although there are numerous methods of symptomatic treatment in insomnia, causative regimens with proven efficacy and safety are still to be established.

Excess body weight is among the major modifiable risk factors for sleep disorders. A reduction in body weight can be associated with a reduction in prevalence of sleep disorders [12]. Therefore, bariatric surgery may be considered an effective, safe method to causatively treat sleep disorders concomitant to excess body weight, and the sustainable effect of bariatric surgery in contrast to other methods should be emphasized.

In a randomized controlled trial conducted by Spaeth et al., short sleep duration was indicated as an independent risk factor for statistically and clinically significant increase in calorie intake and weight gain [15]. There is evidence that excess body weight leads to sleep quality deterioration, reduction in sleep duration, and sleep disturbances, which lead to body mass gain, creating a vicious circle [16]. This positive feedback loop may be one of mechanisms involved in the failure of medical procedures targeting the treatment of obesity. In this context, bariatric surgery seems to be a particularly legitimate method to treat obesity as well as its related sleep disorders.

The direct correlation between OSA and obesity is well established, and there are numerous studies analyzing the influence of bariatric surgery on remission of OSA [9,10,12,13]. However, the association between sleep disorders other than OSA, including insomnia and its symptoms and obesity is not well established. There are few studies on the influence of obesity and the following bariatric treatment on different sleep disorders. Therefore, there is a strong need to evaluate the impact of surgical treatment of obesity on prevalence of sleep disorders and to find whether sleep disorders other than OSA are possibly related to excess body weight.

### Purpose of this Study

The purpose of this study was to assess the influence of laparoscopic sleeve gastrectomy on prevalence and intensity of different sleep disturbances. The primary end-points were remission of insomnia measured by a reduction in Athens Insomnia Scale score and reduction in prevalence of sleep disturbances, including difficulties in falling asleep, night awakenings, early morning awakenings, snoring, and nightmares. The secondary aim of this study was to find a correlation between the excess weight loss percentage (EWL%) after bariatric surgery and changes in sleep quality.

## 2. Materials and Methods

This study was designed as a prospective observational study. A total of 99 patients with obesity qualified for laparoscopic sleeve gastrectomy who came to the Department of General, Oncological, Metabolic, and Thoracic Surgery, Military Institute of Medicine and were invited to participate in this study. All subjects gave their informed written consent before completing the questionnaire. The participants were asked to fill in the same version of questionnaire before surgery and after 6 months of follow-up. The questionnaire is an original tool written by our study group. Nineteen participants failed to complete the follow-up and did not fill in the postoperative survey. The reason for the loss of follow-up was that some of the participants from long-distance places of residence did not come to postoperative visits and therefore there was no possibility for them to complete the follow-up. The final sample included 80 patients, who all completed the preoperative and postoperative survey. The exclusion criteria included minority (age of less than 18 years old), lack of patient consent, and failure to complete the follow-up survey.

The questionnaire used in this study (Supplementary Materials S1) included questions on patients’ basic characteristics (age, height, weight, education, profession) and a set of structured questions about different sleep disturbances, such as difficulties in falling asleep, night awakenings, early morning awakenings, snoring, and nightmares, as well as eating at night and daytime dysfunction.

### 2.1. Athens Insomnia Scale

Additionally, we used an instrument commonly used to evaluate sleep presence and severity of sleep disorders and insomnia—the Athens Insomnia Scale (Supplementary Materials S2). The AIS is a questionnaire based on the *International Classification of Diseases, Tenth Revision* criteria designed for quantitative measurement of the severity of insomnia that includes 8 questions [17]. The AIS is used to evaluate the severity of insomnia both for diagnostic purposes and additionally to assess the efficacy of introduced methods of treatment for insomnia. Each answer is rated from 0 to 3 to evaluate the intensity of each problem, with the total score ranging from 0 to 24. The scale was validated in Poland, and 8 points is considered a cutoff score in the general population, characterized by very good consistency (Cronbach alpha = 0.90) and reliability (test–retest reliability, r2 = 0.92). In our study, we considered an AIS score of 8 or more as equivalent to diagnosis of insomnia, following the Polish validation of the AIS score [18].

### 2.2. Statistical Analysis

Statistical analysis was performed using Statistica 13 (StatSoft. Inc., Tulsa, OK, USA). To handle the data loss, the complete case analysis, listwise deletion was used. Normality of the data was tested with the Shapiro–Wilk test. Mann–Whitney U and Student’s *t* tests were used for quantitative data comparison as required. A two-sided Fisher’s exact test and chi-square test were used for categorical and binary data comparison as required. Biserial correlation was carried out for comparison between continuous and dichotomous variables. *p* value <0.05 was considered significant.

### 2.3. Ethical Considerations

This study was anonymous, performed in accordance with the ethical standards aid down in the 1964 Declaration of Helsinki and its latter amendments (Fortaleza). Participants were informed about the aim of this study and informed consent was obtained from every participant. The approval from the Bioethics Committee of the Military Institute of Warsaw was obtained from code 30/WIM/2021.

## 3. Results

### 3.1. General Characteristics of the Study Group

The mean age of the participants was 39 years old (range: 22–68 years old), with median age of 36 years old. Nineteen participants (23.75%) were male and sixty-one (76.25%) were female. Forty-two patients (52.50%) had higher education, thirty-one (38.75%) had secondary education, and seven (8.75%) had primary education. Sixty-nine participants (86.25%) declared cohabitation with other people and eleven (13.75%) lived alone.

The baseline characteristics of the study group are presented in Table 1.

The mean BMI before surgery was 44.33 kg/m^2^ (range: 31.44–78.90 kg/m^2^), after surgery was 37.45 kg/m^2^ (range: 25.40–61.23 kg/m^2^), the mean difference between pre- and postsurgical BMI was 6.88 kg/m^2^ (CI95 5.76–8.00; *p* < 0.05).

**Figure 1:**
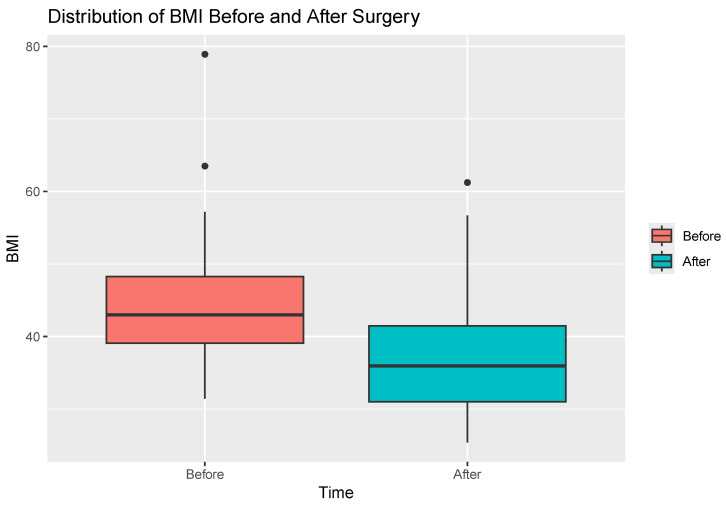
Distribution of BMI before and after surgery.

The distribution of BMI in the study group before and after surgery is presented in Figure 1.

**Figure 2:**
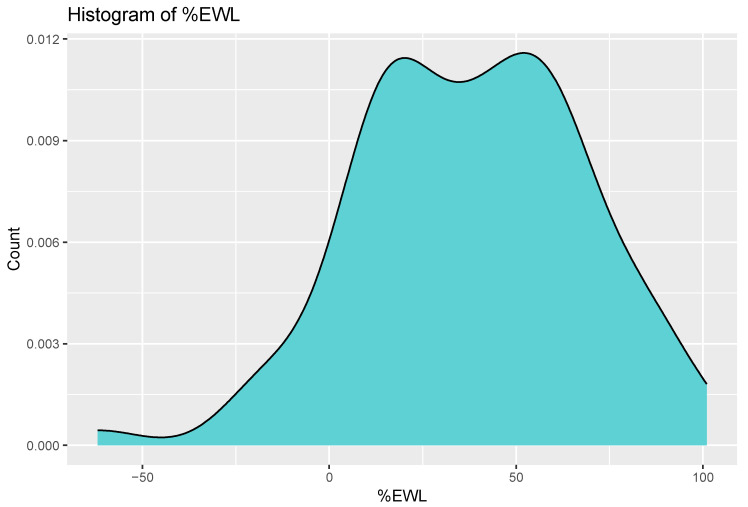
Distribution of EWL%.

The mean EWL% was 37.92%. The distribution of EWL% in the study group is presented in Figure 2.

### 3.2. Sleep Disturbances before and after Surgery

The reduction in incidence of night awakenings was statistically significant, with 40.00% of participants reporting night awakenings before surgery and, respectively, 25.00% after surgery. A significant reduction was also observed in the rate of patients who reported snoring, with 60.00% before the surgery and 38.75% after the surgery (*p* < 0.05).

There were no significant differences found in the prevalence of difficulties in falling asleep before and after surgery, with a reduction from 33.75% to 28.75% (*p* = 0.56), nor in the prevalence of early morning awakenings and nightmares.

There was a biserial correlation present between EWL% and reduction in snoring (*p* < 0.05). No statistically significant correlation was between EWL% and other sleep disturbances, difficulties in falling asleep (*p* = 0.31), night awakenings (*p* = 0.88), early-morning awakenings (*p* = 0.50), and nightmares (*p* = 0.32).

There was no correlation found between sex and changes in prevalence of any sleep disturbances analyzed in this study. There were no significant changes in daily functioning before and after surgery, with 35,00% of participants reporting disturbances in daily functioning before surgery and 28,75% after surgery (*p* = 0.38).

### 3.3. Athens Insomnia Scale before and after Surgery

The reduction in AIS scores was observed both in selected separate questions and in the total AIS scores. The mean total AIS score before surgery was 7.21 and 5.99 after surgery; the change was statistically significant (*p* < 0.05). The total AIS score of 8 or more, a cutoff score for insomnia diagnosis according to the Polish validation of the Athens Insomnia Scale [18], was present in 44.16% of cases before surgery and in 38.00% after surgery (*p* = 0.52).

There was a significant difference in the incidence of awakening during the night score before and after surgery (*p* < 0.05; CI 0.022–0.341), sleep quality (*p* < 0.05; CI 0.0105–0.4311), well-being during the day (*p* < 0.05; CI 0.0273–0.4143), and sleepiness during the day (*p* < 0.05; CI 0.101–0.444).

There were no significant differences found in sleep induction, final awakening, total sleep duration, or daytime functioning capacity scores before and after surgery.

The results of the Athens Insomnia Scale score before and after surgery are presented in Table 2.

**Figure 3:**
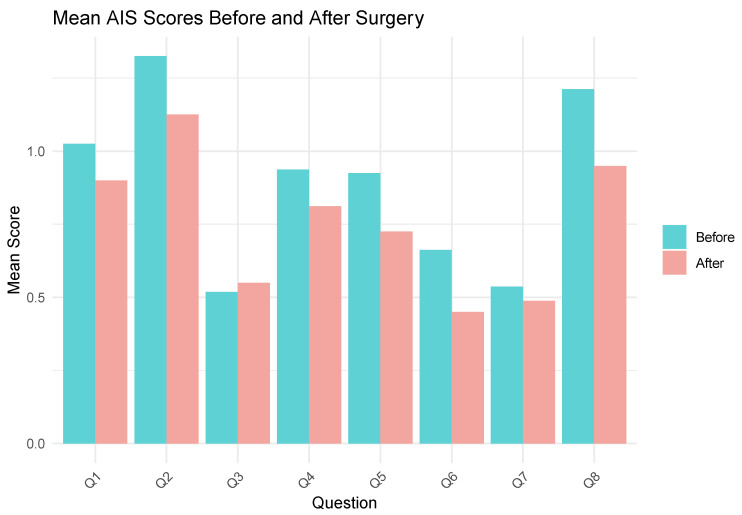
Athens Insomnia Scale scores before and after surgery (Question 1 (Q1)—sleep induction; Q2—awakenings during the night; Q3—final awakening; Q4—total sleep duration; Q5—sleep quality; Q6—well-being during the day; Q7—functioning capacity during the day; Q8—sleepiness during the day).

Mean AIS scores before and after surgery are presented in Figure 3.

No direct correlation was found between EWL% and the level of reduction in AIS score, neither in separate questions, nor in the total AIS score. Then we divided patients into two groups: those for whom there was no amelioration in AIS score observed, neither in individual questions, nor in the total score, and those for whom there was amelioration present, either in separate questions or in total score. Point biserial correlations showed there was a positive relation between some of the questions and total AIS score and EWL%; however, no statistically significant correlation was found (*p* value between 0.07 for awakenings during the night and 0.6 for the total AIS score). The results were parallel when the AIS score changes were analyzed in correlation with BMI changes.

## 4. Discussion

In our study, we found that sleep disturbances other than OSA, including insomnia, have high prevalence in the population of patients with obesity, which is the novelty of our study. Most studies about sleep disorders and obesity are focused only on the prevalence of OSA. However, other sleep disturbances, including difficulties in falling asleep, night awakenings, early morning awakenings, snoring, and nightmares, as well as eating at night and the following daytime dysfunction also present high prevalence in populations with obesity. Patients with sleep disorders suffer from, among many other symptoms, constant fatigue, reduced attention level, lowered mood, and daytime dysfunction and therefore sleep disorders, including insomnia, often underestimated, lead to an important deterioration in quality of life.

We found that the effects of laparoscopic sleeve gastrectomy observed after 6 months of follow-up also included remission of insomnia and reduction in incidence of sleep disturbances. There was a reduced rate of patients reporting difficulties in falling asleep and snoring after bariatric surgery when compared to the same group before surgery. Additionally, the total AIS score was reduced after surgery, as well as some of the separate questions, including awakening during the night, sleep quality, well-being during the day, and sleepiness during the day. We also observed a reduction in the rate of patients who achieved more than 8 points in the AIS score, therefore fulfilling the criteria of insomnia diagnosis, after the surgery. The results of our study lead to some clinical implications. The association between the rate of prevalence of sleep disturbances and obesity suggests that bariatric surgery might be a tool that would allow for reducing the incidence of sleep disturbances and amelioration in quality of life of patients with obesity. The results of our study suggest that insomnia should possibly be considered an obesity-related co-morbidity, and included in the indications for bariatric surgery, as the reduction in the rate of insomnia and other sleep disturbances would improve daytime functioning and the general quality of life of patients with obesity.

The literature on the subject of sleep disturbances other than OSA and the utility of the Athens Insomnia Scale in patients with obesity qualified for bariatric surgery is scarce. Wrzosek et al. analyzed the relation between insomnia, depressive symptoms, and eating habits in patients qualified for bariatric surgery [19]. The study group included 361 patients with obesity. Level of sleep disturbances was measured with the AIS and the Apnea-Hypopnea Index (AHI) was used to assess the presence and severity of OSA. The median score for AIS was found to be 5 [3,4,5,6,7,8] with a range of 0–24, and 47% (171) patients had an AIS score of ≥6, which was considered equivalent to diagnosis of insomnia, according to criteria chosen for the purpose of this study. In our study, the mean total AIS score before surgery was 7.21 and 5.99 after surgery, the change was statistically significant (*p* < 0.05). In our study, we considered AIS score of 8 and more as equivalent to diagnosis of insomnia, a cutoff score for insomnia diagnosis according to the Polish validation of the Athens Insomnia Scale [18]. A total AIS score of 8 or more [18], was present in 44.16% of cases before surgery and in 38.00% after surgery (*p* = 0.52). Due to different cutoff score in Wrzosek’s study, it is not possible to compare the prevalence of insomnia with our study.

Pinto et al. analyzed influence of bariatric surgery on sleep quality in a group of 60 patients [20]. The Pittsburgh Sleep Quality Index (PSQI) and excessive daytime sleepiness by the Epworth Sleepiness Scale (ESS) were used to evaluate the sleep quality at the baseline timepoint and after surgery. This study also included an assessment of OSA risk, measured by the Berlin Questionnaire, and of depressive symptoms, evaluated with the Beck Depression Inventory—Short Form. PSQI score was found to be significantly improved after bariatric surgery (6.4 ± 3.8 versus 4.1 ± 2.8; *p* < 0.001), as well as ESS score (8.1 ± 4.7 versus 6.0 ± 3.3; *p* < 0.001) and Beck Depression Inventory score (9.8 ± 7.0 versus 4.7 ± 4.6; *p* = 0.001). There was an impressive reduction in the risk for OSA, with 68.3% before surgery versus 5% after surgery. The group included 18 patients with a baseline ESS score >10 that indicated excessive daytime sleepiness (EDS), 12 of whom had a normal ESS score after surgery. The conclusion from this study was that bariatric surgery had a beneficial effect both on sleep quality and reduction in daily sleepiness, which is consistent with the results from our study.

Presence of sleep disturbances in individuals with obesity and the relationship between poor sleep and obesity are subject to numerous studies [21,22,23]. Salwen-Deremer et al. searched to establish the prevalence and characteristics of poor sleep of candidates for bariatric surgery [24]. The study included 5427 individuals seeking bariatric surgery and 5180 controls. Analyzed data were abstracted from presurgical self-report questionnaires on sleep quality, insomnia, anxiety, and depression: 40.4% of bariatric group reported 30 min and more to fall asleep, 46.7% indicated sleep time of less than 6.5 h, 65.1% assessed their sleep quality as generally poor, and 30.8% reported clinically significant insomnia symptoms. The sleep efficiency was significantly lower in patients qualified for bariatric surgery than in the controls representing the general population.

Obesity has been reported to be associated with short sleep duration and it may be considered as an independent risk factor for deteriorated sleep quality [25,26,27,28]. O’Halloran et al. analyzed sleep duration and quality in a group of 203 patients with BMI > 35 kg/m^2^, qualified for bariatric surgery, using PSQI [29]. The purpose of the study was to determine whether patients attending for bariatric surgery had poor sleep independent of OSA status. The average sleep duration in bariatric candidates was 6.5 (SD 1.6) hours, therefore shorter than recommended; 67.9% of patients had PSQI scores indicating poor sleep quality (PSQI > 5). There was no significant association between presence of OSA measured by AHI index and sleep quality and duration. The study showed a correlation between BMI and sleep quality (*p* = 0.007) and BMI and PSQI score, with 1 kg/m^2^ weight gain corresponding to a 0.097 decrease in PSQI score. Therefore, a reduction in BMI achieved by means of bariatric surgery leads to amelioration of sleep quality.

Data from the available literature show that sleep duration and quality improve by short-term follow-up after bariatric treatment, but data are scarce on effects during long-term follow-up. In a study by Reid et al., sleep duration was analyzed in correlation with BMI and body composition in 49 patients 3 to 16 years after bariatric surgery. An association was found between an earlier sleep timing midpoint during the weekend and lower BMI [30].

Kline et al. examined the relationship between a composite measure of sleep health and change in weight and body composition among adults in a weight loss intervention to establish the relationship between sleep and attempted weight loss [31]. The study group included 125 individuals with excess weight or obesity who participated in a 12-month behavioral weight loss intervention, with assessment of sleep, weight, fat mass, and fat-free mass at baseline, 6 months, and 12 months. Six sleep dimensions (regularity, satisfaction, alertness, timing, efficiency, and duration) were analyzed and qualified ‘good’ or ‘poor’ using questionnaires and actigraphy to count the total score. Mean baseline and 6-month sleep health was 4.5 ± 1.1 and 4.5 ± 1.2, respectively. Mean weight, fat mass, and fat-free mass changes from 0 to 6 months were −9.3 ± 6.1%, −16.9 ± 13.5%, and −3.4 ± 3.4%, respectively, and 0.4 ± 4.8%, −0.3 ± 10.3%, and 0.7 ± 4.1% from 6–12 months. Better sleep health was associated with greater subsequent weight loss (*p* =0.016) and fat loss (*p* = 0.006), but not fat-free mass loss (*p* = 0.0232). Better sleep health measured by regularity, satisfaction, timing, and efficiency were each associated with weight and/or fat loss (*p* = 0.041).

In our study, we observed amelioration in sleep quality in a group of patients who underwent LSG. Positive influence on sleep quality is also presented in the literature after Roux-en-Y gastric bypass (RYGB) surgery. Vafa et al. aimed to evaluate the impact of RYGB on sleep quality, comparing 25 individuals qualified for bariatric surgery and 29 controls [32]. Participants filled in questionnaires about their sleep quality, anxiety, and depression at baseline and after 12 months. In the bariatric candidates, PSQI score was found to have been decreased from a mean of 7.7 to 3.8 and there was a decrease in prevalence of OSA (*p* < 0.001).

Even though we observed amelioration in sleep quality after bariatric treatment in our study, confirmed also in other studies, persevering deterioration in sleep quality can also be observed in specific groups of postoperative bariatric patients. Lawson et al. analyzed a group of 145 patients 6 months after LSG with eating concerns—loss-of-control (LOC) eating. Deteriorated sleep quality was reported by 58.6% of participants and the decrease was associated with the severity of eating disorder, daily physical and mental functioning, and level of stress, and reversely associated with EWL% [33]. Yannakoulia et al. indicate that sleep quality and duration are positively associated with weight loss management [34].

Factors influencing sleep quality in patients with obesity were analyzed by Eid et al. [35]. The study aimed to find a relationship between weight and sleep quality. The analysis was adjusted for behavior such as night-eating, insufficient physical activity, and alcohol and electronic device use. The study included 161 patients with normal weight, excessive weight and obesity, who filled in online questionnaires about their sleep quality, presence of night-eating, level of physical exercise, alcohol use, electronic device use, anxiety, and depression at baseline and after 3 months. High BMI, adjusted for aforementioned specific behavior, was associated with higher incidence of sleep disturbances at baseline and reduced sleep quality after 3 months. The researchers concluded that there is an association between a patient’s weight and their sleep quality, independent of effects of their co-existing behavior, although co-existing specific behavior may also adversely influence sleep quality.

### Limitations of this Study

A possible limitation of our study may be the recall bias and the subjectivity of patients’ opinions. However, there was no incentive to introduce dishonesty into the responses. Additionally, this study was conducted in one bariatric center, where laparoscopic sleeve gastrectomy is a dominant bariatric surgery procedure, although it is parallel to all bariatric centers in our country. Also, 20% of participants did not complete the follow-up, and the loss of data might have influenced the statistical power of our study, which could have caused bias in the estimation of parameters and reduced the representativeness of the sample. Additionally, as this study was dedicated to only one of the surgical techniques, and the results might have been different if this study had included other surgical techniques. The association between Roux-en-Y gastric bypass and other bariatric procedures with prevalence of sleep disorders in patients with obesity before and after surgery should be further established.

## 5. Conclusions

Laparoscopic sleeve gastrectomy was observed to have a positive effect on insomnia remission in patients with obesity, measured by a significant reduction in Athens Insomnia Scale scores in follow-up 6 months after surgery. Additionally, patients after bariatric surgery reported less night awakenings, and there was a lower rate of snoring. Therefore, bariatric surgery can be considered an effective therapeutic tool for insomnia in patients with obesity. The correlation between EWL% and level of remission in different sleep disturbances is yet to be analyzed in further studies, as is the utility of the Athens Insomnia Scale in patients with obesity qualified for bariatric surgery. Our study was dedicated to analyzing the association between laparoscopic sleeve gastrectomy and the prevalence of different sleep disorders; however, further research should also include other bariatric surgical techniques and their possible correlation with remission of sleep disorders in patients with obesity.

## Figures and Tables

**Table 1:** Baseline characteristics of the study population.

Variable (*n* = 80)	Mean Value	SD
Age [years]	39.00	10.07
Mean BMI before surgery [kg/m^2^]	44.33	7.36
Mean BMI after surgery [kg/m^2^]	37.45	8.07
Male/female, *n* (%)	19/61 (23.75%/76.25%)	
Education:		
-Higher	42 (52.50%)	
-Secondary	31 (38.75%)	
-Primary	7 (8.75%)	
Cohabitation with other people	69 (86.25%)	
Living alone	11 (13.75%)	

*n*: number of patients; BMI: body mass index.

**Table 2:** The results of the Athens Insomnia Scale score before and after surgery.

Question	Before Surgery		After Surgery		*p* Value
	Mean	SD	Mean	SD	
Q1—sleep induction	1.03	0.99	0.90	0.91	0.24
Q2—awakenings during the night	1.33	0.65	1.13	0.66	0.03
Q3—final awakening	0.52	0.68	0.55	0.78	0.59
Q4—total sleep duration	0.94	0.81	0.81	0.77	0.24
Q5—sleep quality	0.93	0.90	0.73	0.75	0.04
Q6—well-being during the day	0.66	0.80	0.45	0.65	0.03
Q7—functioning capacity during the day	0.54	0.64	0.49	0.62	0.44
Q8—sleepiness during the day	1.21	0.63	0.95	0.58	0.01
Total AIS score	7.21	4.20	5.99	3.97	0.02

SD—standard deviation, AIS—Athens Insomnia Scale.

## Data Availability

The data presented in this study are available on request from the corresponding author due to patient privacy.

## Supplementary Materials

The following supporting information can be downloaded at: https://www.mdpi.com/article/10.3390/jcm13164820/s1, Supplementary Materials S1: Questionnaire used in the study. Supplementary Materials S2: Athens Insomnia Scale.
