# Supplementary material for: Sleep Quality and Bariatric Surgery—Can We Treat Sleep Disturbances and Insomnia in Patients with Obesity with Laparoscopic Sleeve Gastrectomy?
Source: J Clin Med. 2024 Aug 15;13(16):4820. doi: 10.3390/jcm13164820 (PMC11355848; doi:10.3390/jcm13164820)
Supplement: Supplementary file 1 [file jcm-13-04820-s001.zip › jcm-3099915-supplementary.pdf]

Supplementary Materials

Supplementary Materials S1 – Questionnaire used in the study

QUESTIONNAIRE

ASSESSMENT OF QUALITY OF SLEEP AND WELL-BEING IN PATIENTS QUALIFIED FOR BARIATRIC SURGERY

Please tick if your answer is positive or fill in the empty boxes.

Date

Day:	Month:	Year:

Personal data

Date of birth:

Day:	Month:	Year:

Height:	Weight:

Marital status:

Do you live:

□ alone

□ with other people

Education:

□ high

□ secondary

□ primary

Profession:

Source of income:

□ professional work

□ sickness benefit

□ pension

□ family allowance

□ maintenance of other people

Sleep disturbances:

□ difficulties in falling asleep

□ night awakenings

□ zbyt wczesne budzenie się rano

□ snoring

□ nightmares

□ reported symptoms were present also in the family

If so, please indicate the affected family members:

The reported symptoms have been present:

□ for less than 1 month

□ for more than 1 month

□ for more than 6 months

□ for more than 1 year

□ since childhood

Supplementary Materials S2 – Athens Insomnia Scale [17]

**Sleep factors**	**Athens Insomnia Scale**			
Sleep induction	0: No problem	1: Slightly delayed	2: Markedly delayed	3: Very delayed or did not sleep at all
Awakenings during the night	0: No problem	1: Minor problem	2: Considerable problem	3: Serious problem or did not sleep at all
Final awakening	0: Not earlier	1: A little earlier	2: Markedly earlier	3: Much earlier or did not sleep at all
Total sleep duration	0: Sufficient	1: Slightly insufficient	2: Markedly insufficient	3: Very insufficient or did not sleep at all
Sleep quality	0: Satisfactory	1: Slightly unsatisfactory	2: Markedly unsatisfactory	3: Very unsatisfactory or did not sleep at all
Well-being during the day	0: Normal	1: Slightly decreased	2: Markedly decreased	3: Very decreased
Functioning capacity during the day	0: Normal	1: Slightly decreased	2: Markedly decreased	3: Very decreased
Sleepiness during the day	0: None	1: Mild	2: Considerable	3: Intense
